# Residential mobility: Towards progress in mobility health research

**DOI:** 10.1177/0309132516649454

**Published:** 2016-05-19

**Authors:** Tim Morris, David Manley, Clive E. Sabel

**Affiliations:** University of Bristol, UK; University of Bristol, UK and OTB, Delft University of Technology, The Netherlands; University of Bristol, UK

**Keywords:** adverse life events, childhood, health behavior, mental health, migration, residential mobility

## Abstract

Research into health disparities has long recognized the importance of residential mobility as a crucial factor in determining health outcomes. However, a lack of connectivity between the health and mobility literatures has led to a stagnation of theory and application on the health side, which lacks the detail and temporal perspectives now seen as critical to understanding residential mobility decisions. Through a critical re-think of mobility processes with respect to health outcomes and an exploitation of longitudinal analytical techniques, we argue that health geographers have the potential to better understand and identify the relationship that residential mobility has with health.

## I Introduction

It has long been hypothesized that there are important and substantial links between an individual’s residential mobility biography (hereafter ‘mobility’) and their health outcomes (Bentham, 1988; Boyle et al., 1999, 2002; Findley, 1988; Strachan et al., 1995). Within the geographical health literature there is a divergence between those studies that take an aggregate population view and those that take a disaggregated individual level perspective, which has produced disparate practices in how mobility is understood and conceptualized. Studies in the aggregate (population migration) literature generally depict mobility as an event often – but not always – associated with the advancement of lifestyle through improvements to housing, neighbourhoods, or employment, for example. This paradigm has been substantiated by the ‘healthy migrant’ effect; at the population level migrants are seen to experience better health than non-migrants (Bentham, 1988; Boyle and Norman, 2009). Within the individual level literature, however, mobility has generally been viewed as a negative and stressful event – a viewpoint substantiated by numerous studies reporting associations between increased rates of mobility and poor health outcomes (Jelleyman and Spencer, 2008; Scanlon and Devine, 2001).

Population level migration approaches have been important for determining broad patterns in mobility and health but, as with all studies of aggregated data, they do not permit inferences to be made at the individual level; to do so risks the ecological fallacy (Robinson, 1950; Subramanian et al., 2009). As such, they neglect much of the exploration and drilling down into the detail and complexity of patterns between mobility and health outcomes. Whilst work that identifies the potential associations between factors is crucial, the advent of better individual level data, improvements in modelling approaches, and developments in (quantitative) theory have moved disciplines forward so that the ‘Holy Grail’ is to develop a better understanding of the complex causal relationships between social exposures and outcomes. In order to disentangle the complex interrelationships between mobility and health, an analytical framework focusing on the individual, their experiences, biography, and detailed histories of their physical and social exposures is now required. Many of the arguments that follow are of relevance to population level migration studies. However, our critique should be viewed as specific to individual level studies encompassing what we have termed the ‘health mobility’ literature.

Outside of the health literature there have been substantial developments that provide context to the moves that individuals make. For instance it is notable that lifecourse perspectives, although not new (Clark and Dieleman, 1996; Elder and Shanahan, 2006; Mulder and Wagner, 1993), have been increasingly prevalent in both the mobility and wider geography literature in recent years (Bailey, 2009; Coulter et al., 2015; Spallek et al., 2011) but appear largely ignored in the health domain.^1^ The lifecourse approach calls for events such as mobility to be considered within the wider perspective of the life cycle rather than abstracted from context at single points in time. Given that mobility is intrinsically linked to stages and events throughout life, such an approach is necessary for a fuller understanding of mobility. Within the discussion that follows, a central pillar to our position is that social health research which focuses on mobility could draw heavily on the resources of the residential mobility literature. We suggest that engaging with a lifecourse focus linked to robust longitudinal analysis and adopting a pragmatic but necessary view of mobility as dependent upon, rather than independent of, a wider set of circumstances is required to move the literature forward. Whereas the health mobility literature focuses exclusively on the event (in this case moving), a lifecourse approach views the pathway to an event as important as the event itself. Yet despite the substantive interest in mobility, the lack of explicit connections between literatures has resulted in a deficiency of theoretical and empirical advances transferring from the residential mobility to the health literature.

There are multiple linkages to other external literatures that should also be acknowledged when unpicking any potential relationship between health and mobility. One of the largest and most important in terms of understanding residential exposures is that of neighbourhood effects (Manley et al., 2013; Van Ham et al., 2012, 2013), a literature that has sought to examine and unpack the ways in which factors intrinsic to neighbourhoods impact upon health outcomes (Dietz, 2002; Diez Roux, 2001, 2003). Within the context of mobility, there is evidence that while moves to similar or less deprived neighbourhoods may follow the healthy migrant rule, moves to more deprived areas are associated with poorer health (Norman et al., 2005; Tampubolon, 2012; Tunstall et al., 2014). Moreover, given that mobility often involves transitions through different neighbourhoods, this literature has a part to play in the assessment of mobility health research; ignoring the contextual information of mobility would be to oversimplify its settings.

Focusing on the lifecourse more explicitly does not deny the spatial element of geography. The neighbourhood effects literature suggests that the contextual influences surrounding both mobility and health must be taken into account alongside temporal variations and the influence of lifecourse events in order to take seriously the complex relationships between people and place, and mobility and health. Furthermore, although we adopt a quantitative approach we recognize that both mixed methods and qualitative approaches can also bring substantial insight to our understanding. In particular the use of mixed methods approaches to ‘nuance the null’ and explain the exception (DeLuca et al., 2012: 208) highlight the complementary way in which both qualitative and quantitative work with longitudinal analysis can substantially further our understanding. A number of the issues raised within this review are broader than the quantitative sub-discipline and are of relevance to critical, qualitative literatures in geography and mobility. However, we restrict the specificities of our arguments below to the quantitative modelling of health and mobility.

This paper proceeds as follows. First we outline mobility and the factors that contribute to the (im)mobility of individuals. Next we provide an overview of a range of individual outcomes that mobility has been linked to in the health literature, with a particular focus on the sub-fields of mental health and health related (‘risky’) behaviours. We then focus on five key limitations that we feel remain prevalent throughout the health mobility literature and inhibit a full and detailed understanding of the health impacts of (im)mobility. Having critically examined the literature, we then draw upon theoretical and methodological advancements from mobility and neighbourhood effects work to develop a framework through which health focused mobility research can advance in order to obtain more robust, appropriately situated results and better inform scientific knowledge on the health impacts of mobility.

## II Residential mobility

Over a century ago Ravenstein examined migration patterns and posited that they were governed by ‘push-pull’ processes; unfavourable living conditions ‘push’ people out of one location while favourable conditions ‘pull’ them into another (Ravenstein, 1889). Whilst this simple view holds (and remains a central tenet to population level migration studies), it ignores a vast range of complexities that are inherent in individual mobility. Key amongst these complexities is the problem that mobility is not a straightforward or uniform process (Lee, 1966; Rossi, 1955) but a complex social issue. It is not simply a process that matches people to homes (Clark et al., 2006), but the result of an ‘outcome of interplay between preferences, resources, opportunities, and constraints’ (Bolt et al., 2009: 505).

Research has uncovered a wide range of individual characteristics and life events that influence mobility which we can consider in three groupings. First are individual characteristics such as age (Bailey and Livingston, 2007; Canfield et al., 2006; Champion, 2005; Khoury et al., 1988); employment (Böheim and Taylor, 1999; Böheim and Taylor, 2002); socio-economic position (Bailey and Livingston, 2005; Brimblecombe et al., 2000); income (Fell et al., 2004); housing tenure (Grundy, 1986); neighbourhood deprivation (Bailey and Livingston, 2007); and marital status (Plewis, 2007). The second group comprise life events that can trigger moves and include childbirth (Kulu, 2005); union formation (Grundy and Fox, 1985; Mulder and Wagner, 1993); union dissolution (Feijten and van Ham, 2010; Flowerdew and Al-Hamad, 2004; Ford, 1997); and changes in employment (Clark, 2005; Shumaker and Stokols, 1982). The third group relate to the cultural and social preferences that individuals consider when moving house such as moving preferences and migration intentions (De Jong, 2000); destination and origin household and neighbourhood satisfaction (Clark and Huang, 2003; Lee et al., 1994; van Ham and Feijten, 2008); and consideration of making ‘positive’ moves upwards on housing (Bolt et al., 2009; Clark et al., 2014) or neighbourhood ladders (Clark et al., 2006).

Coulter and Van Ham (2013) argue that these factors influence mobility through the disequilibrium that arises between needs and resources as a result of changing circumstances. This influence persists throughout the lifecourse but the relative importance of factors varies at different stages (Champion, 2005; Cooke, 2008; Duke-Williams, 2009), influencing the nature, regularity, and consequences of household moves in complex ways. This makes mobility a complicated topic to study as no one characteristic or event is singularly responsible but instead they contribute to form a complex set of influences and interactions on people’s propensity to move. Characteristics and preferences may operate in a more passive way than life events which exert a more active, directly causal influence on mobility – and indeed may even override characteristics entirely (De Groot, 2011; De Groot et al., 2011) – but their influence on mobility cannot be ignored or understated.

The presence of children within the household is an important factor for mobility. While young adults are more likely to move than other age groups, those with children move shorter distances than any other demographic group (Champion, 2005; Clark, 2013; Nivalainen, 2004) in order to minimize social network disruption, provide stability for children, remain within the same labour market areas, or to suffice changing household needs (Kulu, 2005; Varady, 2005). The lack of quantitative research focus on children is intriguing, even more so given the increase of attention to mobility within children’s geographies in the critical, qualitative literature in recent years (Dobson, 2009; Skelton, 2009). Children are more reliant on smaller and closer peer networks than adults and therefore may be more susceptible to the consequences of exogenous environmental or social change. Moreover, given that children are rarely the driver of moves in terms of household preferences and location selection, the relative lack of selection bias could give us important insights when exploring causal mechanisms. Moves may still be made for reasons relating to the child, for example in the case of school enrolment, but because children do not actively seek to move home or initiate the moving process we can view them more as ‘passengers’ and therefore use them to explore the complex place, mobility and health outcomes more readily than adults.

Our understanding of the motives and decisions behind mobility has increased substantially over the last decade with advances in lifecourse theory for mobility,^2^ and we understand better *when* and *why* people move (Coulter et al., 2015). However, these advances have not fed forward to a better understanding of what *happens* to people who move in terms of their health outcomes.

## III The inter-relation between mobility and health

### 1 Associations between mobility and health outcomes

Within the health mobility literature there is an increasing body of evidence demonstrating that moving may have adverse effects on a wide range of health outcomes. For instance, within the mental health domain the evidence generally indicates that compared to people with lower rates of mobility or who are residentially stable, people who have higher rates of mobility experience poorer general mental health and wellbeing (Bures, 2003; Larson et al., 2004; Oishi and Schimmack, 2010; Tunstall and Pickett, 2009); both a greater number and a more serious degree of internalizing and externalizing emotional and behavioural problems (Ackerman et al., 1999; Anderson et al., 2014; Flouri et al., 2013; Simpson and Fowler, 1994; Wood et al., 1993); and higher rates of depression, anxiety, and distress (Bradshaw et al., 2010; Dong et al., 2005; Gilman et al., 2003; Hooper and Ineichen, 1979; Simpson and Fowler, 1994).

Of course, the health literature is not just concerned with deleterious outcomes but also health behaviours. Higher levels of mobility have been linked to increased incidence of smoking (Dong et al., 2005; Lee, 2007); increased alcohol onset and related problems (DeWit, 1998); early initiation of drug use and drug-related problems (DeWit, 1998; Gasper et al., 2010; Lee, 2007); and increased participation in anti-social behaviour (Simpson and Fowler, 1994), deviance (Haynie et al., 2006), violence (Haynie and South, 2005), and more broad criminal activity (Sharkey and Sampson, 2010). There are also gender specific sexual health impacts: females who move have been found to have increased rates of early and premarital sex and teenage pregnancy (Dong et al., 2005; Stack, 1994; Tonnessen et al., 2013) and higher numbers of sexual partners (Baumer and South, 2001) than females who remained residentially stable.

However, the evidence base is not as clear-cut as this would suggest, as some studies have revealed null effects between mobility and health. Similarly, studies examining wellbeing instead of the *absence* of illness have also implied that mobility is not always associated with reductions in wellbeing and within some groups is even associated with increased wellbeing (Bartram, 2013; Nowok et al., 2013). Complicated interaction effects between mobility and personality can confuse findings further; Stoneman and colleagues (1999) found that increased mobility was related to greater behavioural problems, depression, and social isolation only amongst children who scored low on emotionality or were subject to high caregiver conflict.

An important issue often neglected in the health mobility literature and which can serve to confuse findings further is that of selective migration. This has long been identified in the residential mobility and neighbourhood literatures (Boyle and Norman, 2009; Curtis et al., 2009; Manley and Van Ham, 2012; Norman et al., 2005; Oreopoulos, 2003), in which there has been a particular focus on health-selective migration (Connolly et al., 2007; Darlington et al., 2015; Gatrell, 2011; Riva et al., 2011). Because the factor(s) that drive mobility throughout the lifecourse such as age and socio-economic position (Tunstall et al., 2012) are largely the same as the factors that determine the patterning of health outcomes (Davey Smith et al., 1998; Link and Phelan, 1995), the relationship between mobility and health may be spurious and heavily confounded by selective migration or underlying latent differences between groups of people (Bentham, 1988; Jokela, 2014).

### 2 Children’s health outcomes

We may expect differences between child and adult outcomes because children have little influence over mobility decisions and suffer more complete losses of physical social networks than adults, which as a result may make them more vulnerable. Particularly for children, negative life experiences play a critical role in the onset of psychological conditions (Rutter, 1981; Silver et al., 2002) which can have long-term systematic influences that track into later life (Bailey, 2009). Thus it is clear here that within the individual level focused health literature, mobility is being cast either explicitly or implicitly as a negative experience. Household moves can create disorder and disruption that cause stress (Haveman et al., 1991; Popham et al., 2015); negatively impact development and wellbeing (Compas, 1987; Pearlin et al., 1981); lead to emotional and behavioural problems (Conger et al., 1994; Kohen et al., 2008); experiences of isolation (Stubblefield, 1955) and social exclusion (Cole et al., 2006); and the disturbance of social networks and relationships (Bailey and Livingston, 2007; Brett, 1982; Coleman, 1988; Leventhal and Brooks-Gunn, 2003; Pribesh and Downey, 1999; Stokols et al., 1983).

Of course, where moves are seen to have an effect on outcomes this may be a result of the process rather than the event. The stress surrounding an event such as a household move may reduce parental availability and resources, reduce parent-child interaction, and increase unsupportive parenting and maltreatment (Anderson et al., 2014; Waylen et al., 2008). This would make young children particularly susceptible because they are highly dependent on parental attention and resources (Shonkoff and Phillips, 2000). Recent work has determined a link between adverse events in childhood and ‘psychological wear and tear’ in later life that points to plausible *biological* pathways from negative events to ill health (Barboza Solís et al., 2015).

### 3 The impact of distance and neighbourhoods

Geography has been central to health outcomes and geographical modifiers identified as important components of the health mobility relationship. At the population level, short distance moves are generally associated with poorer health than longer distance moves (Boyle et al., 2002). This is surprising if viewed from the context of the health mobility literature: short distance moves tend to be those that enable the maintenance of social networks and do not require the time, cultural or emotional investment of learning a new environment. By contrast a longer distance move – apparently the less deleterious in terms of health – can be far more disruptive and frequently results in the destruction of important social and cultural ties. Here the event of moving itself may not be the most important aspect of the residential change with respect to health outcomes; it may have as much to do with the motive as the actual move. Whilst long distance moves may be more disruptive they are less likely to be made under duress, and more likely to be related to labour market repositioning or other positive relocations. Short distance moves by comparison could be far more heterogeneous and include a greater degree of forced and stressed circumstances (job loss, union dissolution) where people are forced to downgrade their residential and neighbourhood position as well as positive repositioning moves.

We can view the heterogeneity of the shorter distance moves using evidence from the Moving To Opportunity (MTO) study in the US.^3^ Because they used random assignment methods, the MTO studies suffer less bias than standard observational studies, although the findings have been substantially critiqued (Clark, 2008; DeLuca et al., 2012). The outcomes from the MTO work suggest moving from a high to lower poverty neighbourhood has an overall positive long-term effect on multiple aspects of mental health (Ludwig et al., 2012) and behavioural problems (Fauth et al., 2005), particularly for children (Chetty et al., 2015; Kling et al., 2007; Leventhal and Brooks-Gunn, 2003). From these findings we can infer that in certain circumstances mobility may be beneficial to health over the longer term, adding individual level study weight to population level theory. Similar associations between neighbourhood poverty and mental health outcomes have also been observed (in non-experimental work) from the UK (Tunstall et al., 2012) and mainland Europe (Driessen et al., 1998). It has been suggested that the cause behind improved mental health following a move to low poverty neighbourhoods may be due to a reduction in personal stress from moving *away* from disorderly and dangerous neighbourhoods (Kling et al., 2007; Ross et al., 2000), consistent with a ‘residential stress’ model (Lee et al., 1994).

At the neighbourhood level mobility (turnover) is associated with increased prevalence of mental health problems (Matheson et al., 2006; Silver et al., 2002), substance misuse, disorder, and criminal activity (Chaix et al., 2005; Sariaslan et al., 2013; Silver et al., 2002), while residential *stability* in poor or distressed neighbourhoods has been associated with high levels of distress and increased juvenile delinquency (Peeples and Loeber, 1994; Ross et al., 2000). These findings reinforce the importance of geography and the spatial context of mobility; relationships are far too complicated and interwoven with space, time and context to make uniform statements on the effects that mobility has on health.

### 4 Uncertainty over health impacts

Given the heterogeneous findings in the health mobility literature, the key question of whether and what kind of effect mobility has on health outcomes is therefore complex and difficult to answer. The body of evidence indicates that mobility does have an independent association with a range of health outcomes that cannot be explained entirely by selection effects; in short, mobility fundamentally *matters*. However, conflicting evidence and variations throughout the lifecourse caution the interpretation of the evidence and indicate that these associations are complex and subtly tied in to a range of other factors. Due to a number of limitations present throughout the individual level focused health mobility literature, we do not yet have a sufficient handle on the questions of *how* mobility may matter, *why* it matters, *when* it matters and for *whom* the effects (across the continuum from positive to negative) are the greatest. We believe that by overcoming these limitations it will be possible to make advances along these domains.

## IV Key shortcomings within the health mobility literature

Despite increasing interest in the health impacts of mobility, recent developments in the migration and residential mobility literatures have not transferred to the health mobility literature. We observe five key shortcomings for intervention that can be summarized as the categorization of mobility; the importance of time; the nature of moves; the use of sufficient and relevant background data; and the use of appropriate modelling approaches.

### 1 Categorizing mobility

Empirically modelling mobility as a continuous variable is problematic because the number of observations decreases as the number of consecutive moves increases, resulting in low cell counts in extreme (high mobility) groups.^4^ However, the development of categories to effectively report mobility is not theoretically or empirically straightforward, and a simple cut-off is often utilized to group all individuals moving over a certain threshold as being ‘highly mobile’. This leads to an inevitable theoretical problem: how many moves are required and over what time period before an individual counts as being highly mobile? The common cut-offs for defining high mobility employed within the literature are three or four moves (Jelleyman and Spencer, 2008) independent of the temporal period being investigated. Clearly within the space of a 12-month period three or four moves would classify as highly mobile. But does the same classification apply when a temporal window stretches throughout the whole of childhood? These differences are not problematized within the literature, and whilst such categorizations are widely adopted they are often not justified explicitly nor are the implications for analytical outcomes discussed. Stokols and Shumaker (1982) argued that mobility was inadequately conceptualized and that it should be reconceptualized as a more fluent, biographical trajectory through life in order for it to be examined appropriately. Yet 30 years later this reconceptualization has not taken place. It is the story behind each mobility event, and the summation of exposures, contexts and decisions that have been experienced previously by an individual and that contextualize the event, that need to be used in the analysis as well as the event itself. In short, knowing simply that someone is a mover or stayer is insufficient.

Taking this forward, studies employing mobility as a key variable of interest in determining an outcome must not only make clear the theoretical basis for their categorization of mobility but also for the period they examine and its implications. It is not difficult to conceive that multiple moves within a short time period are more likely to be indicative of a chaotic or chronic moving profile than the same number within a long time period, yet studies have been implemented using different ranges to assess the same outcome (DeWit, 1998; Hoffmann and Johnson, 1998) as studies that assess associations between substance use and mobility defined over periods of birth–18 and ages 12–17 respectively.

### 2 The importance of time and timing

Many existing studies use a simplistic view of time from either one of two dominant approaches. The first utilize specific temporal windows in order to examine the effects of mobility at certain life stages such as pre-school (Duncan et al., 1998) or adolescence (Haynie et al., 2006; Lee, 2007). The second utilize broader periods such as childhood or lifetime (DeWit, 1998; Gilman et al., 2003; Oishi and Schimmack, 2010; Verropoulou et al., 2002). Where these studies measure cumulative mobility over these longer periods there is an implicit assumption that time has a constant and uniform effect on outcomes: In other words, it does not matter *when* a move occurred, only *if* a move occurred. However, this assumption is contradicted by evidence from studies that have explicitly investigated temporal trends in mobility. Haveman and colleagues examined mobility in three separate periods in childhood and found evidence that the effects of mobility on academic attainment varied over time (Haveman et al., 1991). Similarly, Rumbold and colleagues examined behavioural trajectories with mobility over three periods to age nine and found evidence that differences in internalizing behaviour only existed amongst children who had moved twice or more between birth and two years (Rumbold et al., 2012). Using broader periods, the studies of Anderson et al. (2014) and Duncan et al. (1998) identified temporal differences in the effect of mobility on behavioural and educational outcomes. Using a focus on the final four years of compulsory education in the UK, Leckie observed strikingly different strength of effects on attainment for mobility by age (Leckie, 2009).

While these studies focus almost exclusively on educational attainment, their findings have implications for health studies that temporal trends should not be ignored and that critical periods of exposure likely exist in physical and socio-emotional development, an idea supported by child development theory (Shonkoff and Phillips, 2000). While a lack of focus on temporal trends and critical periods may be in part driven by data limitations, researchers should pay greater attention to the influence of time and acknowledge that different effects may occur at different times in different people. Neighbourhood effect studies have adopted novel techniques and a biographical approach to mobility (Hedman et al., 2015; van Ham et al., 2014), which, while not on health outcomes, are transferable to the health mobility literature.

### 3 The nature of moves as positive or negative experiences

The overwhelming majority of health mobility studies tend to group all moves together regardless of their context and motivation. Such an approach fails to acknowledge that moves can be positive and bring beneficial changes (employment opportunities, improving housing conditions), or negative and lead to unfavourable or even harmful changes (eviction, loss of financial resources). Furthermore, whether moves are positive or negative may vary between individuals; a positive move for one individual may manifest itself as a negative move for another. This decomposition of the mobility process has not yet been explicitly made in the health mobility literature. Some scholars have acknowledged that moves can be positive as well as negative (Verropoulou et al. 2002; Sharkey and Sampson 2010; Stokols and Shumaker 1982; Ketende et al., 2010; Gasper et al., 2010), but to our knowledge only two individual level studies have explicitly examined the effects of positive and negative moves with a health focus. Blackman and colleagues found that individuals who improved their residential status through relocation away from properties with serious physical defects or unstable neighbourhood environments (i.e. made positive moves) experienced reductions in depression compared to those who remained in such properties (Blackman et al., 2003). Woodhead and colleagues found evidence that residents experiencing displacement (negative moves) subsequently experienced poorer mental health while residents experiencing a desired move did not (Woodhead et al., 2015).

The associational links observed between population health and neighbourhood deprivation (Norman et al., 2005) also implies that the nature of moves must be considered within the neighbourhood as well as the housing context. There is evidence that moving to a more deprived area leads to poorer health outcomes (Exeter et al., 2015; Tunstall et al., 2012, 2014) yet ‘deprivation mobility’ – how neighbourhood deprivation that people are exposed to change as they move from one neighbourhood to another (Boyle et al., 2009) – has not been fully incorporated into the health mobility literature. MTO studies have found that positive mobility away from violent neighbourhoods is associated with improved mental health (DeLuca et al., 2012), but these findings can only be interpreted contextually because individual circumstances were not thoroughly reported. Clearly there may be differential effects between positive and negative moves, and the two should be separated in research.

The individual level health literature also suffers from an underlying assumption that there are one-to-one relationships between staying and good health and moving and bad health – a reductionist view that is in conflict with population level migration studies and implies homogeneity within mobility groups. This has rarely been challenged yet such a dichotomy cannot be correct because residential stability may not be beneficial if people are unable to ‘escape’ an area, as highlighted in some of the studies above. Thus it is not just about the reductionist categories of ‘movers’ and ‘stayers’ but the heterogeneity within those groups that becomes important.

The lack of moving preferences and choices in research studies is a significant contribution to the poor definition of the nature of moves across the health mobility literature. Preferences play a large role in determining the extent to which a move is experienced as positive or negative (Bolt et al., 2009; Coulter and Van Ham, 2013) and so due consideration should be given to the motivating factors (Findlay et al., 2015) and social sorting processes (Sampson, 2008) that drive mobility. The use of moving preferences may permit researchers to disentangle the complex health effects of mobility and explain why it can have different effects on different people in similar circumstances.

### 4 Appropriate data use

Our fourth major limitation within the literature relates to the underuse of data that is required to fully illustrate the circumstances and situation(s) surrounding mobility. Lack of data availability may prevent full analyses of some datasets and play a part in these limitations, but the health mobility literature largely appears to ignore many relevant data in research design, even where they are available. For instance, while demographic characteristics (age, gender, socioeconomic position, and tenure) are well accounted for in most studies, other factors such as life events (those that the literature highlights as the key triggers of mobility) are generally not accounted for. This means that unobserved confounding (omitted variable bias) may bias results, making mobility appear more significant as a causal event than it truly is. Such bias occurs when the effects of an omitted variable (for example divorce) is ‘picked up’ by an intermediate variable (residential mobility), causing the effect of the intermediate variable to be inflated beyond that of its own independent effect (Clarke, 2005; Elwert and Winship, 2014). This is of crucial importance because negative life events are themselves also robustly associated with a wide range of negative health outcomes (Bzostek and Beck, 2011; Conger and Donnellan, 2007; Hoffmann, 2006; Mauldon, 1990), and so the inflation of mobility effects where events are excluded is likely to be significant.

Despite calls to examine events alongside mobility made almost two decades ago (DeWit, 1998), few authors even acknowledge that mobility may be acting as a proxy for unobserved variables such as life events, let alone include them. Flouri and colleagues recently highlighted this problem by stating: ‘researchers do not always pay careful attention to the factors that influence why families move in the first place’ (Flouri et al., 2013). We further this and call attention to the fact that many of the observed ‘independent’ health effects of mobility are at best likely to be misrepresented as they may be demonstrating proxy effects of life events that themselves have a sizeable effect. It is important to be clear here. We are not suggesting that mobility has little or negligible independent effect on health outcomes, but we seek greater clarity on this relationship to better understand the mobility process and draw attention to the fact that mobility may be more of an intermediary factor than a fundamental *cause*.

There is evidence in the literature to support our concern. In some circumstances it has been demonstrated that controlling for a wide range of background factors and life events entirely attenuates the effect of mobility on various outcomes (Dong et al., 2005; Pribesh and Downey, 1999). Furthermore, despite the fact that (negative) life events rarely occur independently of one another (Dong et al., 2004) and may have different effects on mobility when analysed together, those studies that have taken into account life events alongside mobility have tended only to do so with single events (although notable exceptions that have analysed multiple events simultaneously exist; see Clark, 2013; Morris et al., 2015). There are also oversights in the definition of some life events. For example, most studies examining union dissolution fail to discriminate between separation and divorce, despite evidence that they have different effects on mobility (Clark, 2013). By excluding appropriate background information on characteristics and life events that are drivers of mobility, studies risk presenting biased and confounded results. Ultimately, studies must strive to include these data because it is the detailed testing of such information that will lead to a better understanding of mobility.

### 5 Modelling approaches

Our final major limitation relates to modelling approaches utilized in the health mobility literature. In a major review of health outcomes associated with childhood mobility, half of the studies identified were cross-sectional and many over-simplified their analysis (Jelleyman and Spencer, 2008). Because cross-sectional research only examines a ‘snapshot’ of data at a single time point instead of measuring change over time, by definition it ignores the temporal dimension of mobility (Quillian, 2003) and therefore cannot assess effects properly within the wider lifecourse approach that we call for. Because of this, many of the health differences observed by mobility categorizations may be due to characteristics *more common* in mobile families rather than any causal effects of mobility. Such selection effects have been discovered where longitudinal data and advanced analytical techniques have been used (Gasper et al., 2010). Additionally, because of the confounding between certain individual characteristics or events and both mobility and health, cross-sectional research offers no protection against reverse causality – in this case health selective migration. Population level migration studies have demonstrated the importance of selective migration, and therefore utilizing analytical research methods that are unable to account for this is short-sighted.

Given the time lag between life events and mobility and indeed between mobility and health outcomes, effects may not develop instantaneously at the point of the move but develop throughout incubation periods of exposure (Galster, 2012). Popham and colleagues (2015) identified that while people who moved experienced a rise in distress prior to moving, it was not evident until a year after the move. Had their study been cross-sectional and used data that ceased at the point of mobility, this effect would have been missed and their conclusions different. It is clear therefore that the complexities inherent in the process of mobility that this review has highlighted cannot be appropriately modelled with cross-sectional approaches.

It is feasible that the conflicting findings and lack of corroboration of research into the health effects of mobility are in part due to the discordant analytical methods that have been utilized. Across the literature a wide range of mobility categorizations, timescales, preference assumptions, data, and methods have been used; it is not entirely surprising that findings are so dissonant. While the health mobility literature has advanced greatly over the past decades, there is scope for improvement by adopting theory and practice from the migration literature.

## V Developing the health literature: How to move forward

Given the areas for intervention outlined above, we propose a number of directions that the health mobility literature can take in order to improve ascertainment of health outcomes relating to mobility. Making direct linkages between the health, migration, and neighbourhood literatures will allow researchers to better identify health effects of mobility that are more accurate, appropriately situated in context, and robust to confounding (whether observed or unobserved, due to reverse causality or selection bias). A focus on childhood offers theoretical and practical advantages that, coupled with theoretical, methodological, and data advances, can move the literature forward.

### 1 The importance of children

It will be valuable for future health mobility research to adopt a focus on children for a number of reasons. Firstly, a child-specific focus in mobility research has been somewhat under-utilized – a fact that seems odd given that young families with children are the most mobile group of people. Secondly, children are particularly susceptible to damaged networks and environments as a result of moving and have fewer direct positive returns from mobility than adults, for whom mobility may be positive if it brings employment improvements, for example. Thirdly, as (young) children do not have a choice in the mobility patterns of their families, they provide a rare analytical group in which the problems of health selective migration are minimized. It is possible that there may still be bias of selective migration, for example where parents’ ill health transfers to children or where parents have concerns about potential health implications of the surrounding environment, but this will be far smaller than self-selective migration bias when studying adults.

### 2 The lifecourse approach to advance theory

Theoretical advances to overcome the limitations outlined in this review can be made largely by adopting the lifecourse approach. We should acknowledge recent advances towards this within the health and environmental exposure literature on the ‘Exposome’ (Jacquez et al., 2015), but this is very much a nascent literature. A greater focus on mobility as a biography that is taken into account alongside other life events will permit a ‘bigger picture’ view of mobility, one that offers new and more detailed conceptual understandings and contextualizes mobility as a construct of a much larger and complicated system instead of a unique event completely independent of other aspects of life. Researchers must make greater efforts to include more detail on the family environment, the neighbourhood environment, the occurrence and timing of life events, and people’s conditions and preferences. We do not propose a major shift, but a marrying of literatures in order to develop conceptual and methodological approaches and encourage progress in geographical health research.

These advances will permit studies to explore the existence of data-driven trajectories and critical periods or theoretical developmental periods of exposure to mobility, helping to tease out the true health outcomes of such a complex process. The use of broad timescales will help to overcome the problem of discordant periods in the literature, making results immediately more comparable and meaningful. It is important, though, that researchers remain aware that frequent movers are likely to be disproportionately excluded from analysis, simply because tracking becomes more difficult for the studies that provide data the greater the level of participant mobility. The literature suggests that these groups are at the greatest risk of negative health outcomes (Cole et al., 2006), and so the exclusion of these groups may bias results and lead to under- or over-statement of findings. While this cannot be avoided, it is important that researchers give full consideration and a detailed account of the groups that are lost to follow up in longitudinal studies.

It is of vital importance that this focus on the temporal is not made at the expense of geography. Denying the importance of context and geography (whether in spatial or relational terms) is to oversimplify the complex inter-relationships between people and place that form a central pillar of mobility. To ignore geographical context would be to throw out all of the excellent work that has been conducted at the population level by migration studies and risks committing an individualist fallacy. Considering that mobility is a social process that is undoubtedly linked to neighbourhoods, this would be an extremely unwise direction for research to take.

### 3 Longitudinal methodologies to advance methodology

Methodological advances are also required to move the health literature forward. Given the limitations with cross-sectional modelling strategies as outlined above, studies should aim to use longitudinal and panel-modelling approaches that are more appropriate because they suit the analytical challenges that we highlight. Multilevel modelling approaches (Goldstein, 2011) provide appropriate methods for bridging the gap between individual-focused mobility and population-focused migration studies because they can account for patterns at both scales simultaneously. Such approaches can therefore tease out the complex underlying relationships between population movement and health outcomes and may help to identify whether the difference in findings between these literatures (and, as a result, the difference in conceptualization of mobility as a positive or negative event) reflects the phenomenon of Simpson’s paradox (Blyth, 1972) or true substantive differences. While they are no panacea, longitudinal and multilevel approaches are less prone to bias than cross-sectional models, can appropriately handle time, change, and selection effects, and can explicitly model the differences between the causal effects of mobility and the underlying differences between groups of people. As such, their use will allow a more rigorous and robust testing of hypotheses than cross-sectional approaches.

We echo a recent call in this journal for greater understanding of mobility through longitudinal analysis (Coulter et al., 2015) and add to this call that there is a great need for *appropriate* longitudinal analytical methods. In order to determine change in health status, separate within and between person effects, and identify causal processes, longitudinal multilevel models are necessary. However, mobility patterns also need to be examined in a far richer and more detailed manner than the crude categorizations that currently persist in research design; where people move multiple times in studies, more complex analytical methods may have to be deployed, particularly if finer details such as the length of exposure to particular events and contexts are to be taken into account (see Van Ham et al. (2014) for a deprivation exposure example). Models are no more or less than abstractions of a reality based on a set of ‘partial truths about reality’ (Baumol, 1992: 55) that we have chosen to accept as being sufficient to allow insight into the complexities of individual developments. As such, it is crucial to recall that no modelling procedure can overcome fundamentally flawed theoretical conceptualizations or give insight beyond the extent of the original data. Given the fluidity of mobility there is scope for researchers to adopt a wider range of analytical methods to tease out casual health inferences instead of using over-simplified or purely associational analysis. However, these methods must be made use of by researchers; too commonly, studies using longitudinal sources of data are under-specified with a cross-sectional approach.

### 4 The use of appropriate datasets to overcome data limitations

In order to accommodate these theoretical and methodological advances certain data advances may also be required. After all, a lack of data richness has historically restricted researchers from being able to adopt a more detailed methodology (Long, 1992). An occasion or ‘wave’ based measure of mobility is required in order for temporal trends and critical periods to be identified and examined, meaning that datasets which offer only measures such as lifetime moves are unsuitable. Similarly, multiple measurements of outcomes are required from data if researchers are to overcome the problems of measuring *change* in variables of interest and therefore the true magnitude of effects. Detailed neighbourhood information is a further data requirement that researchers should look to. This is necessary to consider the geographical clustering of individual health phenomena, and to obtain accurate effects at both the individual and contextual level. This assessment of context is theoretically important as people from the same area are more similar to each other than those from other areas (Merlo et al., 2006). Qualitative data also has a role to play in health mobility research as it can help to elucidate in more detail the reasons people have for moving, and better understand the complex relationships between mobility and health outcomes. We acknowledge that the limitations outlined earlier may be driven by data restrictions and, as such, it may not be possible for researchers to overcome *all* of these limitations simultaneously, but given the increase in availability of detailed longitudinal studies we believe that this defence no longer holds across all limitations. Where studies suffer one of the limitations we outline above, researchers should make clear the underlying cause of the limitation. Such a simple but important change would bring added clarity to findings and allow the research community to more easily identify the structural limitations that hinder progress.

## VI Towards progress in mobility health research

There is a vast body of literature linking mobility and adverse health outcomes. Whilst much is well developed with respect to health outcomes, the treatment of mobility has been more limited and characterized by a lack of nuance for understanding a highly heterogeneous process. By acknowledging the residential mobility literature more explicitly, in particular the idea of the lifecourse and mobility biography, we call for a new agenda in health mobility research that advances our understanding of the pathways and linkages between childhood mobility and later health outcomes. Simultaneous advances in life course theory, longitudinal methodology, and resourceful data use will allow us to delve deeper into the complex ways in which mobility influences health outcomes for different people in different situations and better advance understanding of the ‘*what happens’* to people who move.

Developing such an understanding is critical in enabling us to frame the extent to which policies and interventions may address health issues and reduce structural inequalities in society. Research focusing on individuals has indicated that mobility generally has a negative effect on a range of personal mental health and health behaviour outcomes in childhood and later life, but key limitations which persist throughout the literature caution against the accuracy and validity of findings and therefore our understanding of the long-term health impacts of mobility. The limitations that we have discussed highlight the problem caused by the disparate data, methods, and time periods that exist within the health literature. A move to a more standard, thoroughly grounded, well explained and justified approach can help focus the literature to a more coherent and informative future.

Residential mobility health research is at a crossroads. It either continues along its current path of discordant methods and theory, or links in theoretical and methodological advances from other literatures in order to advance. Such a linking will permit the adoption of a new standard that can push the conceptual landscape of the field and more reliably inform academic thinking and public policy.

## Notes

1. This refers specifically to mobility studies. Lifecourse epidemiology has a considerable history but its main focus has been on biological or broad social exposures rather than specific events that occur within the social domain.

2. There is more to the lifecourse than the events we discuss in this paper (Elder and Shanahan, 2006), but we call only upon the events that specifically relate to mobility in the same manner as Bailey (2009).

3. The MTO was a semi-randomized housing mobility experiment sponsored by the US Department of Housing and Urban Development which offered rental assistance housing vouchers to randomly-selected residents in high poverty neighbourhoods with which they could move to low poverty neighbourhoods. See de Souza Briggs et al. (2010) for further details.

4. This categorization problem does not, of course, arise in studies that utilize a crude measure of ‘movers’ vs ‘non-movers’ but, given the heterogeneity of both groups, such studies have a different range of problems associated with them.
